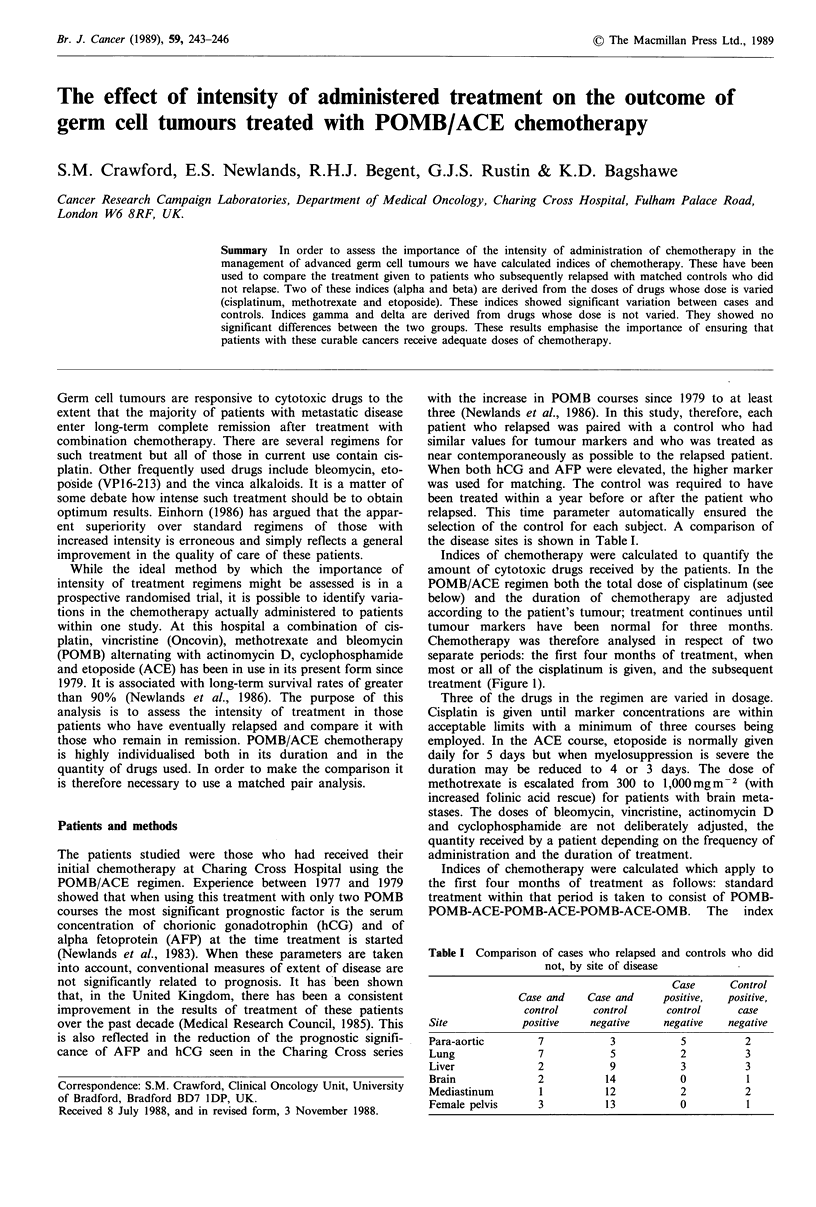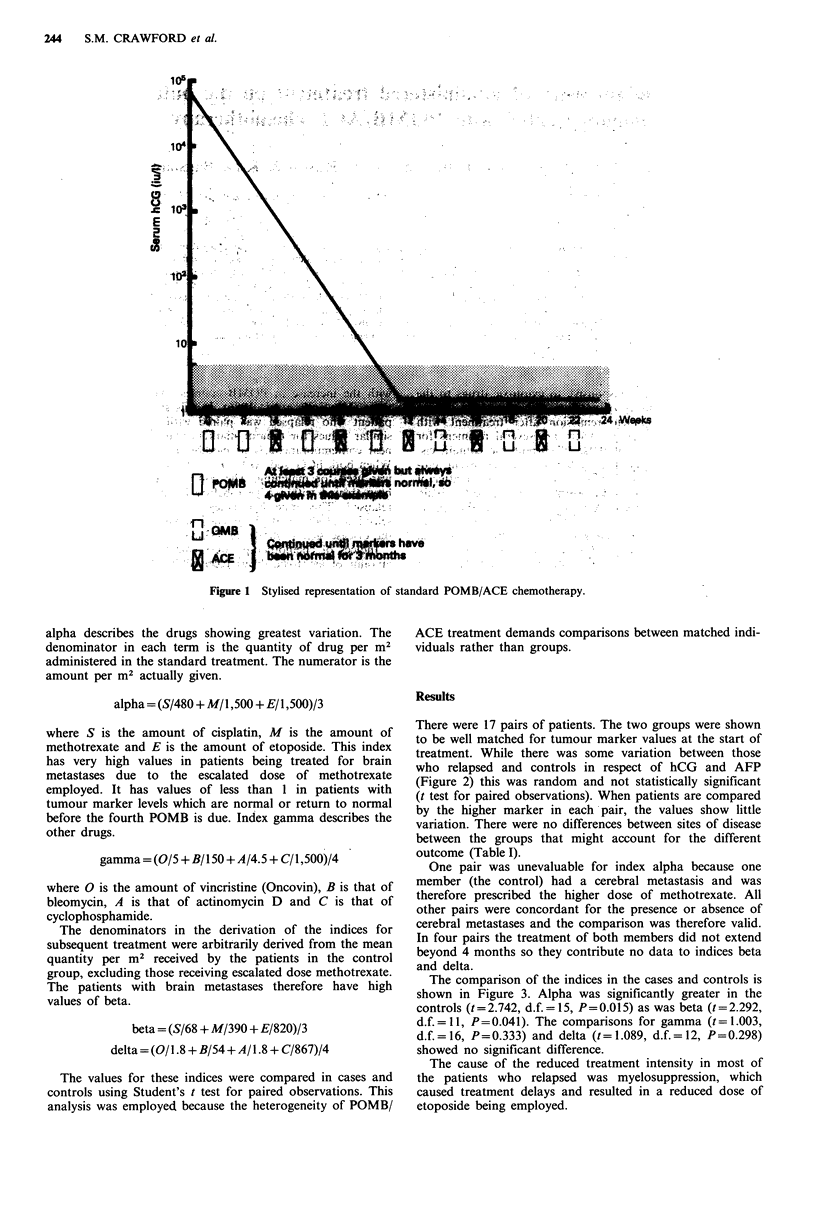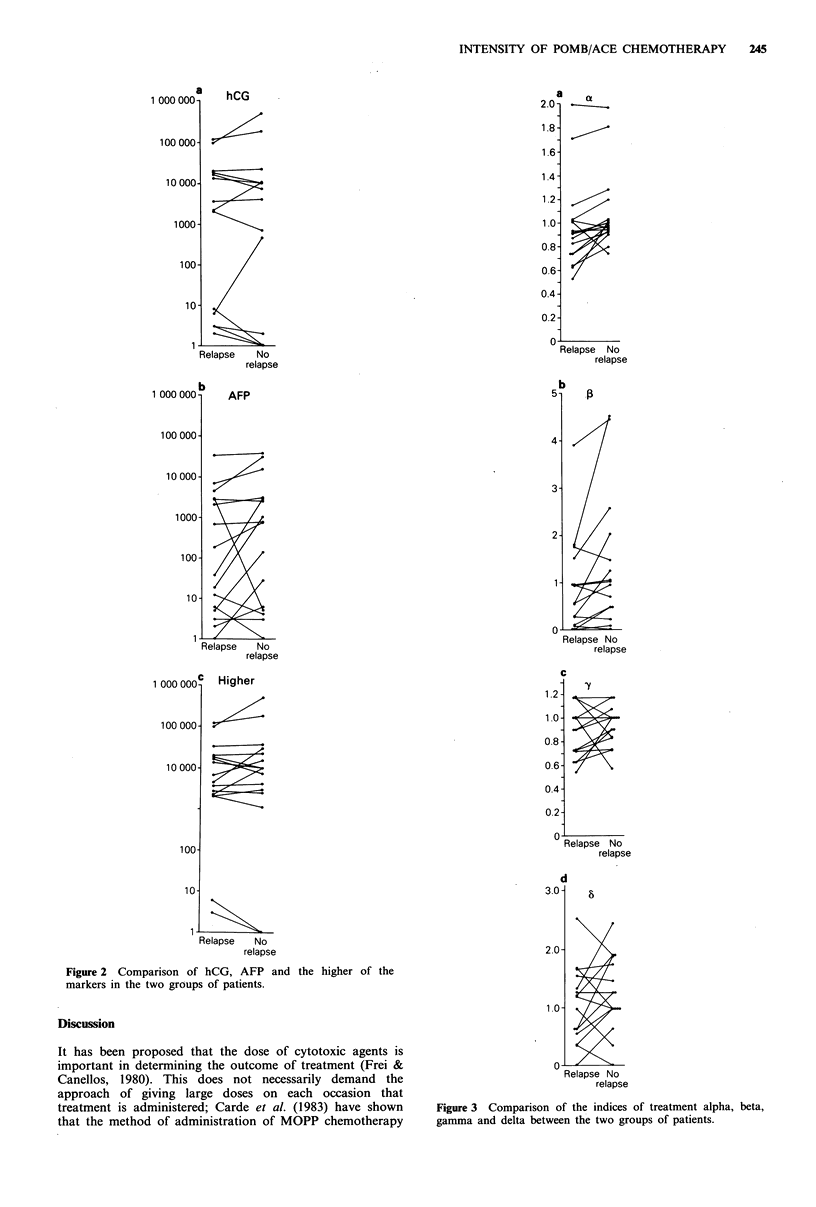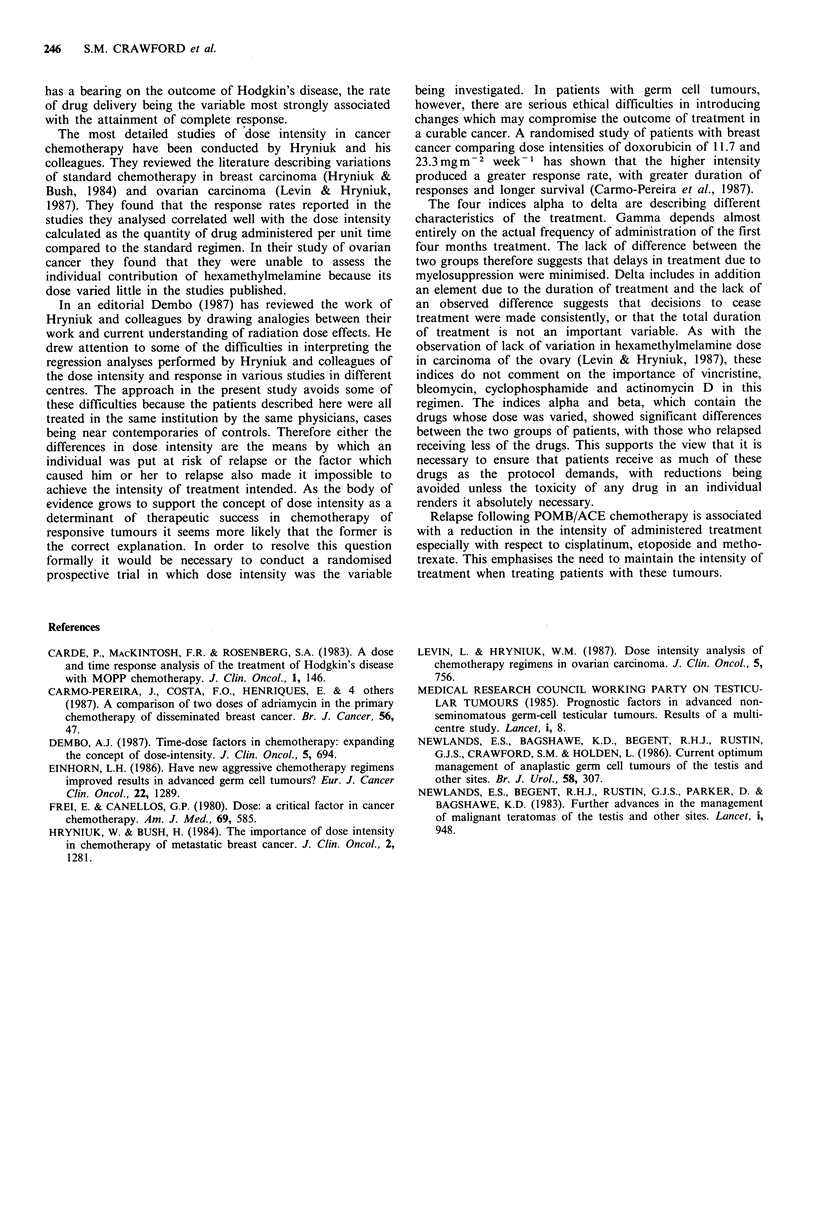# The effect of intensity of administered treatment on the outcome of germ cell tumours treated with POMB/ACE chemotherapy.

**DOI:** 10.1038/bjc.1989.49

**Published:** 1989-02

**Authors:** S. M. Crawford, E. S. Newlands, R. H. Begent, G. J. Rustin, K. D. Bagshawe

**Affiliations:** Cancer Research Campaign Laboratories, Department of Medical Oncology, Charing Cross Hospital, London, UK.

## Abstract

In order to assess the importance of the intensity of administration of chemotherapy in the management of advanced germ cell tumours we have calculated indices of chemotherapy. These have been used to compare the treatment given to patients who subsequently relapsed with matched controls who did not relapse. Two of these indices (alpha and beta) are derived from the doses of drugs whose dose is varied (cisplatinum, methotrexate and etoposide). These indices showed significant variation between cases and controls. Indices gamma and delta are derived from drugs whose dose is not varied. They showed no significant differences between the two groups. These results emphasise the importance of ensuring that patients with these curable cancers receive adequate doses of chemotherapy.


Br. J. Cancer (1989), 59, 243–246 © The Macmillan Press Ltd., 1989

### The effect of intensity of administered treatment on the outcome of germ cell tumours treated with POMB/ACE chemotherapy

S.M. Crawford, E.S. Newlands, R.H.J. Begent, G.J.S. Rustin & K.D. Bagshawe

*Cancer Research Campaign Laboratories, Department of Medical Oncology, Charing Cross Hospital, Fulham Palace Road, London W6 8RF, UK.*

**Summary** In order to assess the importance of the intensity of administration of chemotherapy in the management of advanced germ cell tumours we have calculated indices of chemotherapy. These have been used to compare the treatment given to patients who subsequently relapsed with matched controls who did not relapse. Two of these indices (alpha and beta) are derived from the doses of drugs whose dose is varied (cisplatinum, methotrexate and etoposide). These indices showed significant variation between cases and controls. Indices gamma and delta are derived from drugs whose dose is not varied. They showed no significant differences between the two groups. These results emphasise the importance of ensuring that patients with these curable cancers receive adequate doses of chemotherapy.

Germ cell tumours are responsive to cytotoxic drugs to the extent that the majority of patients with metastatic disease enter long-term complete remission after treatment with combination chemotherapy. There are several regimens for such treatment but all of those in current use contain cisplatin. Other frequently used drugs include bleomycin, etoposide (VP16-213) and the vinca alkaloids. It is a matter of some debate how intense such treatment should be to obtain optimum results. Einhorn (1986) has argued that the apparent superiority over standard regimens of those with increased intensity is erroneous and simply reflects a general improvement in the quality of care of these patients.

While the ideal method by which the importance of intensity of treatment regimens might be assessed is in a prospective randomised trial, it is possible to identify variations in the chemotherapy actually administered to patients within one study. At this hospital a combination of cisplatin, vincristine (Oncovin), methotrexate and bleomycin (POMB) alternating with actinomycin D, cyclophosphamide and etoposide (ACE) has been in use in its present form since 1979. It is associated with long-term survival rates of greater than 90% (Newlands *et al.*, 1986). The purpose of this analysis is to assess the intensity of treatment in those patients who have eventually relapsed and compare it with those who remain in remission. POMB/ACE chemotherapy is highly individualised both in its duration and in the quantity of drugs used. In order to make the comparison it is therefore necessary to use a matched pair analysis.

### Patients and methods

The patients studied were those who had received their initial chemotherapy at Charing Cross Hospital using the POMB/ACE regimen. Experience between 1977 and 1979 showed that when using this treatment with only two POMB courses the most significant prognostic factor is the serum concentration of chorionic gonadotrophin (hCG) and of alpha fetoprotein (AFP) at the time treatment is started (Newlands *et al.*, 1983). When these parameters are taken into account, conventional measures of extent of disease are not significantly related to prognosis. It has been shown that, in the United Kingdom, there has been a consistent improvement in the results of treatment of these patients over the past decade (Medical Research Council, 1985). This is also reflected in the reduction of the prognostic significance of AFP and hCG seen in the Charing Cross series with the increase in POMB courses since 1979 to at least three (Newlands *et al.*, 1986). In this study, therefore, each patient who relapsed was paired with a control who had similar values for tumour markers and who was treated as near contemporaneously as possible to the relapsed patient. When both hCG and AFP were elevated, the higher marker was used for matching. The control was required to have been treated within a year before or after the patient who relapsed. This time parameter automatically ensured the selection of the control for each subject. A comparison of the disease sites is shown in Table I.

Correspondence: S.M. Crawford, Clinical Oncology Unit, University of Bradford, Bradford BD7 1DP, UK.
Received 8 July 1988, and in revised form, 3 November 1988.

Indices of chemotherapy were calculated to quantify the amount of cytotoxic drugs received by the patients. In the POMB/ACE regimen both the total dose of cisplatinum (see below) and the duration of chemotherapy are adjusted according to the patient's tumour; treatment continues until tumour markers have been normal for three months. Chemotherapy was therefore analysed in respect of two separate periods: the first four months of treatment, when most or all of the cisplatinum is given, and the subsequent treatment (Figure 1).

Three of the drugs in the regimen are varied in dosage. Cisplatin is given until marker concentrations are within acceptable limits with a minimum of three courses being employed. In the ACE course, etoposide is normally given daily for 5 days but when myelosuppression is severe the duration may be reduced to 4 or 3 days. The dose of methotrexate is escalated from 300 to 1,000 mg m$^{-2}$ (with increased folinic acid rescue) for patients with brain metastases. The doses of bleomycin, vincristine, actinomycin D and cyclophosphamide are not deliberately adjusted, the quantity received by a patient depending on the frequency of administration and the duration of treatment.

Indices of chemotherapy were calculated which apply to the first four months of treatment as follows: standard treatment within that period is taken to consist of POMB-POMB-ACE-POMB-ACE-POMB-ACE-OMB. The index

**Table I** Comparison of cases who relapsed and controls who did not, by site of disease

| *Site* | *Case and control positive* | *Case and control negative* | *Case positive, control negative* | *Control positive, case negative* |
|---|---|---|---|---|
| Para-aortic | 7 | 3 | 5 | 2 |
| Lung | 7 | 5 | 2 | 3 |
| Liver | 2 | 9 | 3 | 3 |
| Brain | 2 | 14 | 0 | 1 |
| Mediastinum | 1 | 12 | 2 | 2 |
| Female pelvis | 3 | 13 | 0 | 1 |

**Figure 1** Stylised representation of standard POMB/ACE chemotherapy.

alpha describes the drugs showing greatest variation. The denominator in each term is the quantity of drug per m² administered in the standard treatment. The numerator is the amount per m² actually given.

$$\text{alpha} = (S/480 + M/1{,}500 + E/1{,}500)/3$$

where $S$ is the amount of cisplatin, $M$ is the amount of methotrexate and $E$ is the amount of etoposide. This index has very high values in patients being treated for brain metastases due to the escalated dose of methotrexate employed. It has values of less than 1 in patients with tumour marker levels which are normal or return to normal before the fourth POMB is due. Index gamma describes the other drugs.

$$\text{gamma} = (O/5 + B/150 + A/4.5 + C/1{,}500)/4$$

where $O$ is the amount of vincristine (Oncovin), $B$ is that of bleomycin, $A$ is that of actinomycin D and $C$ is that of cyclophosphamide.

The denominators in the derivation of the indices for subsequent treatment were arbitrarily derived from the mean quantity per m² received by the patients in the control group, excluding those receiving escalated dose methotrexate. The patients with brain metastases therefore have high values of beta.

$$\text{beta} = (S/68 + M/390 + E/820)/3$$

$$\text{delta} = (O/1.8 + B/54 + A/1.8 + C/867)/4$$

The values for these indices were compared in cases and controls using Student's $t$ test for paired observations. This analysis was employed because the heterogeneity of POMB/ACE treatment demands comparisons between matched individuals rather than groups.

### Results

There were 17 pairs of patients. The two groups were shown to be well matched for tumour marker values at the start of treatment. While there was some variation between those who relapsed and controls in respect of hCG and AFP (Figure 2) this was random and not statistically significant ($t$ test for paired observations). When patients are compared by the higher marker in each pair, the values show little variation. There were no differences between sites of disease between the groups that might account for the different outcome (Table I).

One pair was unevaluable for index alpha because one member (the control) had a cerebral metastasis and was therefore prescribed the higher dose of methotrexate. All other pairs were concordant for the presence or absence of cerebral metastases and the comparison was therefore valid. In four pairs the treatment of both members did not extend beyond 4 months so they contribute no data to indices beta and delta.

The comparison of the indices in the cases and controls is shown in Figure 3. Alpha was significantly greater in the controls ($t=2.742$, d.f. $=15$, $P=0.015$) as was beta ($t=2.292$, d.f. $=11$, $P=0.041$). The comparisons for gamma ($t=1.003$, d.f. $=16$, $P=0.333$) and delta ($t=1.089$, d.f. $=12$, $P=0.298$) showed no significant difference.

The cause of the reduced treatment intensity in most of the patients who relapsed was myelosuppression, which caused treatment delays and resulted in a reduced dose of etoposide being employed.

**Figure 2** Comparison of hCG, AFP and the higher of the markers in the two groups of patients.

### Discussion

It has been proposed that the dose of cytotoxic agents is important in determining the outcome of treatment (Frei & Canellos, 1980). This does not necessarily demand the approach of giving large doses on each occasion that treatment is administered; Carde *et al.* (1983) have shown that the method of administration of MOPP chemotherapy

**Figure 3** Comparison of the indices of treatment alpha, beta, gamma and delta between the two groups of patients.

has a bearing on the outcome of Hodgkin's disease, the rate of drug delivery being the variable most strongly associated with the attainment of complete response.

The most detailed studies of dose intensity in cancer chemotherapy have been conducted by Hryniuk and his colleagues. They reviewed the literature describing variations of standard chemotherapy in breast carcinoma (Hryniuk & Bush, 1984) and ovarian carcinoma (Levin & Hryniuk, 1987). They found that the response rates reported in the studies they analysed correlated well with the dose intensity calculated as the quantity of drug administered per unit time compared to the standard regimen. In their study of ovarian cancer they found that they were unable to assess the individual contribution of hexamethylmelamine because its dose varied little in the studies published.

In an editorial Dembo (1987) has reviewed the work of Hryniuk and colleagues by drawing analogies between their work and current understanding of radiation dose effects. He drew attention to some of the difficulties in interpreting the regression analyses performed by Hryniuk and colleagues of the dose intensity and response in various studies in different centres. The approach in the present study avoids some of these difficulties because the patients described here were all treated in the same institution by the same physicians, cases being near contemporaries of controls. Therefore either the differences in dose intensity are the means by which an individual was put at risk of relapse or the factor which caused him or her to relapse also made it impossible to achieve the intensity of treatment intended. As the body of evidence grows to support the concept of dose intensity as a determinant of therapeutic success in chemotherapy of responsive tumours it seems more likely that the former is the correct explanation. In order to resolve this question formally it would be necessary to conduct a randomised prospective trial in which dose intensity was the variable being investigated. In patients with germ cell tumours, however, there are serious ethical difficulties in introducing changes which may compromise the outcome of treatment in a curable cancer. A randomised study of patients with breast cancer comparing dose intensities of doxorubicin of 11.7 and 23.3 $mg\,m^{-2}$ $week^{-1}$ has shown that the higher intensity produced a greater response rate, with greater duration of responses and longer survival (Carmo-Pereira *et al.*, 1987).

The four indices alpha to delta are describing different characteristics of the treatment. Gamma depends almost entirely on the actual frequency of administration of the first four months treatment. The lack of difference between the two groups therefore suggests that delays in treatment due to myelosuppression were minimised. Delta includes in addition an element due to the duration of treatment and the lack of an observed difference suggests that decisions to cease treatment were made consistently, or that the total duration of treatment is not an important variable. As with the observation of lack of variation in hexamethylmelamine dose in carcinoma of the ovary (Levin & Hryniuk, 1987), these indices do not comment on the importance of vincristine, bleomycin, cyclophosphamide and actinomycin D in this regimen. The indices alpha and beta, which contain the drugs whose dose was varied, showed significant differences between the two groups of patients, with those who relapsed receiving less of the drugs. This supports the view that it is necessary to ensure that patients receive as much of these drugs as the protocol demands, with reductions being avoided unless the toxicity of any drug in an individual renders it absolutely necessary.

Relapse following POMB/ACE chemotherapy is associated with a reduction in the intensity of administered treatment especially with respect to cisplatinum, etoposide and methotrexate. This emphasises the need to maintain the intensity of treatment when treating patients with these tumours.

### References

CARDE, P., MacKINTOSH, F.R. & ROSENBERG, S.A. (1983). A dose and time response analysis of the treatment of Hodgkin's disease with MOPP chemotherapy. *J. Clin. Oncol.*, **1**, 146.

CARMO-PEREIRA, J., COSTA, F.O., HENRIQUES, E. & 4 others (1987). A comparison of two doses of adriamycin in the primary chemotherapy of disseminated breast cancer. *Br. J. Cancer*, **56**, 47.

DEMBO, A.J. (1987). Time-dose factors in chemotherapy: expanding the concept of dose-intensity. *J. Clin. Oncol.*, **5**, 694.

EINHORN, L.H. (1986). Have new aggressive chemotherapy regimens improved results in advanced germ cell tumours? *Eur. J. Cancer Clin. Oncol.*, **22**, 1289.

FREI, E. & CANELLOS, G.P. (1980). Dose: a critical factor in cancer chemotherapy. *Am. J. Med.*, **69**, 585.

HRYNIUK, W. & BUSH, H. (1984). The importance of dose intensity in chemotherapy of metastatic breast cancer. *J. Clin. Oncol.*, **2**, 1281.

LEVIN, L. & HRYNIUK, W.M. (1987). Dose intensity analysis of chemotherapy regimens in ovarian carcinoma. *J. Clin. Oncol.*, **5**, 756.

MEDICAL RESEARCH COUNCIL WORKING PARTY ON TESTICULAR TUMOURS (1985). Prognostic factors in advanced non-seminomatous germ-cell testicular tumours. Results of a multicentre study. *Lancet*, **i**, 8.

NEWLANDS, E.S., BAGSHAWE, K.D., BEGENT, R.H.J., RUSTIN, G.J.S., CRAWFORD, S.M. & HOLDEN, L. (1986). Current optimum management of anaplastic germ cell tumours of the testis and other sites. *Br. J. Urol.*, **58**, 307.

NEWLANDS, E.S., BEGENT, R.H.J., RUSTIN, G.J.S., PARKER, D. & BAGSHAWE, K.D. (1983). Further advances in the management of malignant teratomas of the testis and other sites. *Lancet*, **i**, 948.